# A Practical Anthropometric Model Incorporating Calf Circumference to Estimate Appendicular Lean Mass in Women with Systemic Lupus Erythematosus

**DOI:** 10.3390/muscles5030048

**Published:** 2026-07-02

**Authors:** Dai Qiyun, Yoshinari Matsumoto, Masao Katsushima, Ryu Watanabe, Yuya Fujita, Shinsuke Yamada, Daiki Habu, Motomu Hashimoto

**Affiliations:** 1 Department of Nutrition, Graduate School of Human Life and Ecology, Osaka Metropolitan University, Osaka 536-8525, Japan; sn22344d@st.omu.ac.jp (D.Q.); habu@omu.ac.jp (D.H.); 2 Department of Clinical Immunology, Osaka Metropolitan University Graduate School of Medicine, Osaka 545-8585, Japan; m22647i@omu.ac.jp (M.K.); ryu.watanabe@omu.ac.jp (R.W.); n24517m@omu.ac.jp (Y.F.); s.yamada@omu.ac.jp (S.Y.); motomuhashimoto@gmail.com (M.H.)

**Keywords:** systemic lupus erythematosus, appendicular lean mass, anthropometry, calf circumference, sarcopenia, prediction equation, skeletal muscle mass index

## Abstract

Accurate assessment of appendicular lean mass (ALM), an essential component for evaluating sarcopenia and nutritional status, typically requires dual-energy X-ray absorptiometry (DXA); however, its widespread clinical application is limited by cost and accessibility. This study aimed to develop a simple anthropometry-based equation for estimating ALM in women with systemic lupus erythematosus (SLE) and to compare its predictive performance with existing models. This cross-sectional study included 92 female patients with SLE. A multiple regression model was developed, incorporating height, body weight, calf circumference (CC), and age. Model performance was internally validated using five-fold cross-validation, and agreement with DXA-measured ALM was assessed using out-of-fold predictions. The diagnostic performance for detecting low skeletal muscle mass index (SMI) was also evaluated and compared with previously published equations (Hwang and Santos). The predicted ALM showed good correlation with measured ALM (R^2^ = 0.76) and moderate agreement (Lin’s concordance correlation coefficient [CCC] = 0.829), with a root mean square error of 1.38 kg. Sensitivity and specificity for detecting low SMI were 65.0% and 88.9%, respectively. The proposed equation demonstrated comparable or superior performance (CCC: Hwang 0.782; Santos 0.672) and may serve as a practical tool for estimating ALM in female patients with SLE.

## 1. Introduction

Systemic lupus erythematosus (SLE) is a multisystem autoimmune disease characterized by the production of autoantibodies against nuclear antigens, immune complex deposition, and chronic inflammation in target organs, such as the skin, joints, and kidneys [1]. Glucocorticoids (GCs) are widely used to treat SLE; however, their adverse effects include glucocorticoid-induced muscle atrophy [2]. Furthermore, some patients develop lupus myositis [3], which may contribute to the progression of sarcopenia by reducing muscle mass and strength.

Sarcopenia is a muscle disease characterized by the progressive and generalized loss of skeletal muscle mass and function, and is associated with an increased risk of falls, physical dysfunction, frailty, and mortality [4]. Low skeletal muscle mass has been reported in patients with SLE, even in relatively young populations [5], highlighting the importance of early assessment and intervention [4]. The skeletal muscle mass index (SMI), which is based on appendicular lean mass (ALM), is widely used to assess sarcopenia [6,7,8]. ALM is typically measured using dual-energy X-ray absorptiometry (DXA) [7,8] and commonly used in clinical research as a practical surrogate for appendicular skeletal muscle mass (AMS) [9,10]. In addition, the Global Leadership Initiative on Malnutrition (GLIM) criteria, an international consensus for diagnosing malnutrition, emphasize the assessment of reduced muscle mass as a phenotypic criterion [11]. Therefore, accurate evaluation of ALM is essential for the diagnosis of sarcopenia and malnutrition, as well as for guiding appropriate therapeutic interventions. Assessment of ALM and skeletal muscle mass typically requires imaging or device-based techniques, such as DXA, bioelectrical impedance analysis, magnetic resonance imaging, and computed tomography [12]. However, these methods have limitations in routine clinical use owing to radiation exposure and requirements for specialized expertise, time, and cost [13,14]. To address these challenges, several anthropometry-based equations for estimating ALM and skeletal muscle mass have been developed [9]; however, most of these equations have been derived from general populations, and studies specifically targeting patients with SLE remain limited. Furthermore, in patients with SLE, whose body composition may be altered by chronic inflammation and pharmacological treatments, the applicability of existing equations has not been sufficiently validated.

This study aimed to develop an ALM estimation equation using clinically accessible anthropometric measurements in women with SLE and to evaluate the applicability of existing ALM prediction equations in this population.

## 2. Materials and Methods

### 2.1. Study Population

This study was a cross-sectional study, conducted between 9 February 2023 and 31 May 2024 among patients with SLE attending the Department of Clinical Immunology, Osaka Metropolitan University Hospital [5]. The eligibility criteria were as previously reported [5]: (1) a diagnosis of SLE based on the 2019 European Alliance of Associations for Rheumatology/American College of Rheumatology (2019 EULAR/ACR SLE) classification criteria [15], and (2) independence in activities of daily living, with the ability to undergo physical function assessments and communicate effectively. Because this study aimed to estimate ALM from anthropometric measurements and to consider the influence of sex, only female patients with SLE who had complete anthropometric measurement data and ALM assessment were included in the analysis (*n* = 92). This study was approved by the Ethics Committee of Osaka Metropolitan University Hospital (approval number: 2022-149; approval date: 4 January 2023). The study was conducted in accordance with the Declaration of Helsinki, and written informed consent was obtained from all participants before enrollment.

### 2.2. Patient Characteristics

Information on patient characteristics, including age, medication use, and SLE disease duration, was extracted from medical records. Smoking status and alcohol consumption were assessed using questionnaires. SLE disease activity was evaluated using the Systemic Lupus Erythematosus Disease Activity Index (SLEDAI) [16], and cumulative organ damage was assessed using the Systemic Lupus International Collaborating Clinics/American College of Rheumatology Damage Index (SDI) [17].

### 2.3. Measurement of Appendicular Lean Mass and Definition of Low SMI

ALM was assessed using DXA (HOLOGIC Horizon W, FA-00247, TOYO MEDIC Co., Ltd., Tokyo, Japan) in whole-body mode. ALM was calculated as the sum of lean mass in the upper and lower limbs. The skeletal muscle mass index (SMI) was calculated by dividing ALM by height squared (m^2^). Based on the Asian Working Group for Sarcopenia (AWGS) 2019 criteria, applicable at the time of the study, low SMI was defined as <5.4 kg/m^2^ [8].

### 2.4. Clinical and Anthropometric Assessments

Anthropometric measurements, including height, body weight, and calf circumference (CC), were performed by trained registered dietitians according to standardized procedures, as previously reported [5]. Height and body weight were measured using a standard stadiometer and scale (AD-6350, A&D Medical Co., Ltd., Tokyo, Japan), and recorded to the nearest 0.1 cm and 0.1 kg, respectively. Body mass index (BMI) was calculated as body weight (kg) divided by height squared (m^2^). The CC was measured with the participant in a seated position, with the knee flexed at 90°, at the site of the maximum circumference of the non-dominant calf on a plane perpendicular to the long axis of the lower leg. Measurements were obtained using an insertion tape (Abbott Japan Co. Ltd., Tokyo, Japan) and recorded to the nearest 0.1 cm. No participant was considered to have clinically significant lower-limb edema that would interfere with CC measurement.

### 2.5. Statistical Analysis

Data are presented as mean (standard deviation). Statistical analyses were performed using IBM SPSS Statistics (version 29; IBM Corp., Armonk, NY, USA), R version 4.5.1 (The R Foundation for Statistical Computing, Vienna, Austria) [18], and MedCalc version 23.1.6 (MedCalc Software Ltd., Ostend, Belgium).

To develop a clinically applicable model for estimating ALM using routinely obtainable variables, a multiple linear regression model was constructed using the forced-entry method in female patients with SLE who had available DXA measurements, with ALM as the dependent variable and height, body weight, CC, and age as the independent variables. To evaluate the internal validity of the model, five-fold cross-validation was performed. In each fold, four subsets were used for training and one subset for validation, and out-of-fold predictions were generated for each participant. The agreement between the predicted and DXA-measured ALM was assessed using these out-of-fold predictions. Model performance was evaluated by calculating the coefficient of determination (R^2^), root mean square error (RMSE), mean absolute error (MAE), and Lin’s concordance correlation coefficient (CCC) [19]. In addition, Bland–Altman analysis was performed to assess systematic bias and the distribution of errors between the predicted and measured ALM, and the mean difference (bias) and 95% limits of agreement were calculated [20]. Furthermore, the skeletal muscle mass index (SMI) was calculated by dividing predicted ALM by height squared (m^2^), and the diagnostic performance for low SMI was evaluated using SMI derived from DXA-measured ALM as the reference standard. Sensitivity, specificity, positive likelihood ratio, negative likelihood ratio, positive predictive value, and negative predictive value were calculated. Agreement between DXA-based classification and equation-based classification of low SMI was assessed using Cohen’s κ coefficient [21]. After cross-validation, a final estimation equation was developed using the full dataset with the same independent variables. For the final model, regression coefficients, standard errors, 95% confidence intervals, *p*-values, adjusted R^2^, standard error of the estimate (SEE), and variance inflation factors (VIF) were calculated. Residual normality was assessed using a Q–Q plot, and homoscedasticity was evaluated using a scatter plot of standardized residuals versus standardized predicted values. In addition, previously reported equations by Hwang et al. [22] and Santos et al. [10] were used for comparative analysis (Supplementary Table S1).

## 3. Results

### 3.1. Baseline Characteristics

The baseline characteristics of 92 female patients with SLE are presented in Table 1. The mean age was approximately 49 years, and the mean disease duration was approximately 20 years. A total of 20 patients (21.7%) were classified as having low SMI.

### 3.2. Development of the Prediction Equation for ALM

A multiple linear regression model was constructed with ALM as the dependent variable, and height, body weight, CC, and age as independent variables (Table 2). The final model was developed using the full dataset, and the resulting equation was as follows: ALM (kg) = −19.493 + 0.120 × height (cm) + 0.131 × weight (kg) + 0.232 × CC (cm) + 0.014 × age (years).

The adjusted R^2^ of the final model was 0.758, and the SEE was 1.26 kg. In addition, the VIF for all variables was <3.4, indicating no evidence of multicollinearity. No major violations of model assumptions were observed.

Internal validation using five-fold cross-validation showed that the coefficient of determination (R^2^) was 0.719, the mean RMSE was 1.38 kg, and the MAE was 1.07 kg.

### 3.3. Agreement Between Predicted and Measured ALM

**Figure 1:**
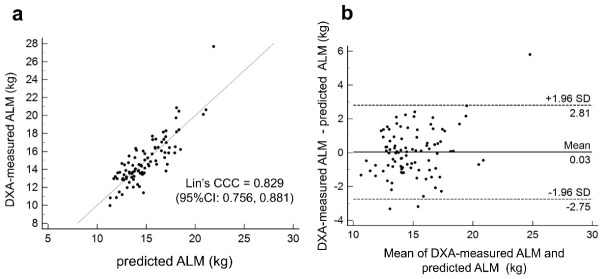
Agreement between predicted and DXA-measured appendicular lean mass (ALM) in female patients with SLE. (**a**) Scatter plot showing the relationship between predicted ALM and DXA-measured ALM. Agreement was evaluated using Lin’s concordance correlation coefficient (CCC). (**b**) Bland–Altman plot showing the difference between predicted and DXA-measured ALM against their mean. The solid line represents the mean difference (bias), and the dashed lines indicate the 95% limits of agreement (mean ± 1.96 standard deviations).

The agreement between predicted and DXA-measured ALM was evaluated using out-of-fold predictions (Figure 1). Lin’s CCC was 0.829, indicating substantial agreement.

Bland–Altman analysis was performed to assess the distribution of differences between predicted and measured values. The mean difference (bias) was small, and most data points were within the 95% limits of agreement, indicating no apparent systematic bias.

### 3.4. Diagnostic Performance for Low SMI

The diagnostic performance of low SMI based on the constructed equation is presented in Table 3. Using DXA measurements as the reference standard, the proposed equation showed high specificity and negative predictive value, indicating a good ability to exclude non-low SMI. In contrast, sensitivity was moderate. In addition, agreement between DXA-based classification and equation-based classification was moderate, with a Cohen’s κ coefficient of 0.53.

### 3.5. Comparison with Existing Prediction Equations

**Figure 2:**
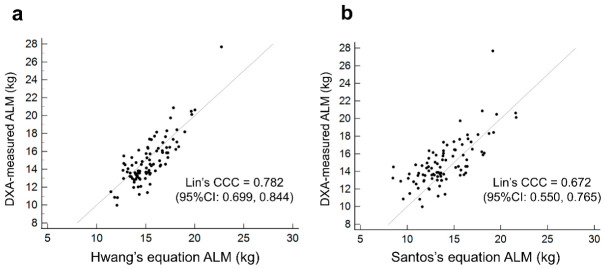
Agreement between appendicular lean mass (ALM) estimated using previously reported equations and DXA-measured values in female patients with SLE. (**a**) Hwang’s equation; (**b**) Santos’s equation.

For comparison with existing equations, the prediction equations proposed by Hwang et al. [22] and Santos et al. [10] were applied to the anthropometric data of the study participants, and agreement between predicted and DXA-measured ALM was evaluated (Figure 2). The CCC was 0.782 for the equation proposed by Hwang et al. [22], and 0.672 for the equation proposed by Santos et al. [10].

Scatter plots show the relationship between ALM estimated using the equations reported by Hwang et al. [22] (a) and Santos et al. [10] (b) and DXA-measured ALM. Agreement was evaluated using Lin’s concordance correlation coefficient (CCC).

## 4. Discussion

In this study, we developed a simple equation to estimate ALM in women with SLE using clinically accessible variables (height, body weight, CC, and age). The proposed equation showed good agreement with DXA-measured values and demonstrated stable predictive performance during cross-validation. In the Bland–Altman analysis, the mean difference was 0.03 kg, indicating minimal bias, and the 95% limits of agreement ranged from −2.75 to 2.81 kg. The differences were generally evenly distributed, and no apparent systematic bias was observed. In an additional exploratory analysis, we compared patients in the highest quartile of absolute prediction error with the remaining patients; however, no significant differences in clinical or anthropometric characteristics were observed, and no clear pattern associated with inaccurate prediction was identified. Furthermore, SMI calculated using the proposed equation showed high specificity and negative predictive value for identifying low SMI.

The proposed equation has high clinical utility because it enables the estimation of ALM without the need for specialized equipment. CC was retained as an independent predictor in the final model. Because CC is a simple anthropometric measure recommended by the AWGS as a case-finding tool for sarcopenia [7,8], its inclusion may enhance the clinical applicability of the proposed equation. It may be particularly useful in settings where DXA is not readily available, and can be used to assess sarcopenia and malnutrition. In addition, given its high specificity and negative predictive value, this equation may be useful for excluding low SMI. Because sensitivity was moderate (65.0%), some individuals with low SMI may not be identified using this approach alone. Its clinical applicability should be interpreted with caution until external validation is performed in independent SLE populations. Furthermore, if validated in external populations, it may be considered an alternative approach for assessing muscle mass within the framework of the GLIM criteria. In addition, the proposed equation may be useful for longitudinal monitoring of muscle mass in routine outpatient care. Because the required variables are routinely collected in clinical practice and do not require specialized equipment or advanced technical skills, the model may facilitate the early identification of patients at risk of low muscle mass, support nutritional assessment and intervention, and be readily implemented by various healthcare professionals, including nurses and dietitians.

In comparison with existing equations, the model by Hwang et al. [22] showed relatively high agreement, whereas the model proposed by Santos et al. [10] showed lower agreement. The equation developed by Hwang et al. [22] was based on an Asian population and included female participants with broadly comparable muscle-related anthropometric characteristics, including calf circumference, appendicular skeletal muscle mass, and skeletal muscle mass index. These similarities may have contributed to the relatively good agreement observed in the present study. In contrast, the equation by Santos et al. [10] was developed for the general population in the United States, with a relatively low proportion of Asian individuals, and differences in body size and fat distribution may have reduced its applicability to the present study population. Existing body composition prediction equations are typically developed for specific populations, and it has been reported that their predictive accuracy may decline when applied to different populations [23]. Furthermore, because both models were developed for the general population, the present findings suggest that body composition prediction equations may perform differently when applied to patients with SLE, whose body composition may be influenced by chronic inflammation and pharmacological treatments.

In patients with SLE, abnormalities in body composition, including reduced muscle mass, have been reported to occur frequently due to chronic inflammation and GC therapy [24,25]. Furthermore, in our previous study, cumulative GC exposure and hydroxychloroquine use were associated with sarcopenia in patients with SLE [5]. These population-specific characteristics may not be fully captured by existing equations developed in the general population and may partly explain why the equation developed in this study showed greater applicability. In addition, patients with SLE frequently experience fatigue, reduced physical activity, and long-term exposure to glucocorticoids, all of which may influence body composition. Such disease-related factors may contribute to body composition patterns that differ from those observed in the general population. Therefore, our findings support the development of population-specific prediction models to improve the accuracy of ALM estimation in patients with SLE.

This study has some limitations. First, this was a single-center, cross-sectional study, and caution is required when generalizing these findings. However, ALM was assessed using DXA, the gold standard for measuring ALM, which may have contributed to the reliability of the results. Second, external validation was not performed, and further studies are required to confirm the validity of the proposed equation. Although the model demonstrated stable performance during internal cross-validation, external validation remains necessary to further evaluate its robustness and generalizability. In addition, caution is warranted when applying the equation outside the range of anthropometric characteristics observed in the present study. Third, this study included only female patients with SLE; therefore, its applicability to male patients with SLE remains unclear. Fourth, the equation included only height, body weight, CC, and age as predictor variables. Although disease activity, cumulative glucocorticoid exposure, and immunosuppressive therapy may influence body composition in patients with SLE, these variables were intentionally excluded to maintain the simplicity and practicality of the model. Future studies should investigate whether incorporating such clinical factors improves predictive performance. Given that an ASM prediction equation incorporating waist circumference has been reported [26], future studies should consider developing ALM estimation equations that include additional anthropometric measurements.

The ALM estimation equation developed in this study may be useful for assessing muscle mass in women with SLE, based on simple anthropometric measurements. Further validation in external populations is required to confirm the clinical applicability of this method.

## 5. Conclusions

In this study, we developed a simple equation to predict DXA-measured ALM based on anthropometric variables, including CC, in patients with SLE and evaluated its validity. Furthermore, the proposed equation demonstrated better performance compared with existing CC-based equations. This equation may be useful for assessing muscle mass in women with SLE.

## Figures and Tables

**Table 1:** Patient characteristics (*n* = 92).

Age (years)	48.9 (14.3)
Height (cm)	156.9 (6.1)
Weight (kg)	53.1 (10.0)
BMI (kg/m^2^)	21.6 (3.7)
Calf circumference (cm)	34.2 (3.4)
ALM (kg)	15.0 (2.6)
SMI (kg/m^2^)	6.1 (0.9)
Prevalence of low SMI (%)	20 (22)
Current smoker (%) (date missing = 1)	14 (15)
Current drinkers (%)	38 (41)
Disease duration (years)	19.5 (9.7)
SLEDAI	2.5 (3.0)
SDI	1.6 (1.8)
GCs (mg/day)	5.0 (3.1)
GCs cumulative dose (g)	50.0 (33.6)
GC user (%)	85 (92)
HCQ user (%)	45 (49)
TAC user (%)	27 (29)
AZP user (%)	12 (13)
MMF user (%)	13 (14)
Bio-ts-/DMARDs user (%)	18 (20)

Dates are shown as numbers (%), average (standard deviation). Abbreviations: ALM, Appendicular lean mass; AZP: azathioprine; BMI, Body mass index; Bio-tsDMARDs, biologic and targeted synthetic disease-modifying antirheumatic drugs; GCs, Glucocorticoids; HCQ, Hydroxychloroquine; MMF, mycophenolate mofetil; SDI, Systemic Lupus International Collaborating Clinics/American College of Rheumatology Damage Index; SLEDAI, Systemic Lupus Erythematosus Disease Activity Index; SMI, Skeletal muscle mass index; TAC, tacrolimus.

**Table 2:** Final prediction equation for appendicular lean mass in female SLE patients.

Predictor	B Coefficient	Standard Error	*p* Value
Intercept	−19.493	4.414	<0.001
Height (cm)	0.120	0.025	<0.001
Weight (kg)	0.131	0.025	<0.001
Calf circumference (cm)	0.232	0.073	0.002
Age (years)	0.014	0.011	0.21

ALM (kg) = −19.493 + 0.120 × height (cm) + 0.131 × weight (kg) + 0.232 × CC (cm) + 0.014 × age (years). ALM was measured by DXA. The final model was constructed using all female participants (*n* = 92). The final model showed an adjusted R^2^ of 0.758 with a standard error of estimate of 1.26 kg. All variance inflation factors were <3.4. Model performance evaluated by 5-fold cross-validation (R^2^, RMSE, MAE reported in text). ALM, appendicular lean mass; CC, calf circumference; DXA, dual-energy X-ray absorptiometry; RMSE, root mean square error; MAE, mean absolute error; SLE, systemic lupus erythematosus.

**Table 3:** Agreement and diagnostic performance of the proposed equation for low SMI classification.

Item	Value
Contingency table (DXA vs. proposed equation)	
True positive (DXA low SMI/predicted low SMI)	13
False negative (DXA low SMI/predicted non–low SMI)	7
False positive (DXA non–low SMI/predicted low SMI)	8
True negative (DXA non–low SMI/predicted non–low SMI)	64
Agreement and diagnostic performance	
Cohen’s κ	0.53
Sensitivity (%)	65.0
Specificity (%)	88.9
Positive likelihood ratio (PLR)	5.85
Negative likelihood ratio (NLR)	0.39
Positive predictive value (PPV, %)	61.9
Negative predictive value (NPV, %)	90.1

κ indicates Cohen’s kappa coefficient. DXA, dual-energy X-ray absorptiometry; SMI, skeletal muscle mass index.

## Data Availability

The datasets used and/or analyzed during the current study are available from the corresponding author upon reasonable request.

## Supplementary Materials

The following supporting information can be downloaded at: https://www.mdpi.com/article/10.3390/muscles5030048/s1. Supplementary Table S1. Existing equations for estimating ALM.
