# Supplementary material for: A Practical Anthropometric Model Incorporating Calf Circumference to Estimate Appendicular Lean Mass in Women with Systemic Lupus Erythematosus
Source: Muscles. 2026 Jul 2;5(3):48. doi: 10.3390/muscles5030048 (PMC13398237; doi:10.3390/muscles5030048)
Supplement: Supplementary file 1 [file muscles-05-00048-s001.zip › muscles-4328107-supplementary.pdf]

Supplementary Table 1 Existing equations for estimating ALM

Predictor	Hwang et al. (Ref.1)	Santos et al. (Ref.2)
Intercept	-8.734	-10.427
Height (cm)	0.148	-
Weight (kg)	0.097	-
Calf circumference (cm)	0.147	0.768
Sex	-3.973(men=1, women=2)	7.523
Age (years)	-0.028	-0.029
Ethnicity	-	see footnote

Ethnicity (Santos et al.): White = 0; Black = 2.203; Mexican American = -0.540; Other = -0.402.

ALM, appendicular lean mass

[1] Hwang, A.C.; Liu, L.K.; Lee, W.J.; Peng, L.N.; Chen, L.K. Calf circumference as a screening instrument for appendicular muscle mass measurement. *J. Am. Med. Dir. Assoc.* **2018**, *19*, 182–4.

[2] Santos, L.P.; Gonzalez, M.C.; Orlandi, S.P.; Bielemann, R.M.; Barbosa-Silva, T.G.; Heymsfield, S.B. New prediction equations to estimate appendicular skeletal muscle mass using calf circumference: Results from NHANES 1999–2006. *JPEN J. Parenter. Enteral Nutr.* **2019**, *43*, 998–1007.
